# The role of thermal niche selection in maintenance of a colour polymorphism in redback salamanders (*Plethodon cinereus*)

**DOI:** 10.1186/1742-9994-3-10

**Published:** 2006-07-05

**Authors:** Erin E Petruzzi, Peter H Niewiarowski, Francisco B-G Moore

**Affiliations:** 1 Department of Biology, University of Akron, Akron, OH 44325-3908, USA

## Abstract

**Background:**

In eastern North America two common colour morphs exist in most populations of redback salamanders (*Plethodon cinereus*). Previous studies have indicated that the different morphs may be adapted to different thermal niches and the morphological variation has been linked to standard metabolic rate at 15°C in one population of *P. cinereus*. It has therefore been hypothesized that a correlated response to selection on metabolic rate across thermal niches maintains the colour polymorphism in *P. cinereus*. This study tests that hypothesis.

**Results:**

We found that the two colour morphs do sometimes differ in their maintenance metabolic rate (MMR) profiles, but that the pattern is not consistent across populations or seasons. We also found that when MMR profiles differ between morphs those differences do not indicate that distinct niches exist. Field censuses showed that the two colour morphs are sometimes found at different substrate temperatures and that this difference is also dependent on census location and season.

**Conclusion:**

While these morphs sometimes differ in their maintenance energy expenditures, the differences in MMR profile in this study are not consistent with maintenance of the polymorphism via a simple correlated response to selection across multiple niches. When present, differences in MMR profile do not indicate the existence of multiple thermal niches that consistently mirror colour polymorphism. We suggest that while a relationship between colour morph and thermal niche selection appears to exist it is neither simple nor consistent.

## Background

Maintenance of variation in populations is a fundamental topic in evolutionary ecology [1]. Mechanisms that maintain variation can be selective, non-selective or a mixture of both. Previous studies have identified factors that maintain colour polymorphisms in many taxa (e.g. [2-6]). However, the factors explaining the coexistence of two common morphs of the Redback Salamander,*Plethodon cinereus *(Plethodontidae) remain poorly understood despite considerable study [7-11].

Natural selection can maintain variation in numerous ways [3,12]. For instance variation can be maintained across multiple niches where different variants are favoured in different microhabitats or at different times [13-15]. Predation, parasitism, competition or sexual selection could result in the maintenance of variation through frequency or density-dependent selection [16-19]. Non-selective processes could also maintain variation. Frequencies of selectively neutral polymorphisms will fluctuate across populations via genetic drift and will generally lead to fixation in some populations [20]. If neutral variation is pleiotropically or chromosomally linked to traits under selection, then the neutral variation could also be maintained via selection on those traits [18,21].

*P. cinereus *are fully terrestrial, lungless, woodland salamanders, which exist as three relatively distinct colour morphs, of which two are commonly found. The striped (redback) morph has a reddish dorsal stripe that extends from the base of the head to the tail, darkly pigmented sides, and a mottled black and white venter. The black (leadback or unstriped) morph is identical to the striped morph except that it has a darkly pigmented dorsum. This variation is heritable, appears to be polygenic in nature [9,22], and is not known to be environmentally plastic. While the variation is polygenic, it is not quantitative since the unstriped morph is qualitatively distinguished by changes in the cellular architecture of chromatophores including a complete loss of erythrophores [23].

The two common colour morphs exist in most *P. cinereus *populations in eastern North America [24]. The relative rarity of monomorphic populations throughout most of the range suggests that genetic drift alone is unlikely to maintain this widespread polymorphism. Previous studies have found that the frequency of the two morphs sometimes varies with climate either geographically [7], or temporally [8,10], indicating that the different morphs may be adapted to different thermal niches. Colour phenotype has also been linked to standard metabolic rate (SMR) at 15°C in one population of *P. cinereus *[11]. Because SMR is an estimate of the maintenance costs that represent a large fraction of an ectotherm's energy budget [25,26] such linkage of colour and SMR provides a mechanism by which the polymorphism might be maintained. This mechanism assumes that differences in metabolic rate translate to differences in fitness since the energy available for survival and reproduction depends on the energy remaining after maintenance costs are paid [25,27].

The purpose of this study was to test a current hypothesis that the colour polymorphism in *P. cinereus *could be maintained via a correlated response to thermal niche selection on metabolic rate [11]. Although a link between metabolic rate and colour morph is known to exist in one population [11], demonstrating the existence of distinct thermal niches requires that each morph specialize on a particular temperature. Only then would thermal niche specialization maintain the polymorphism. This study tested for differences in optimal thermal niche between the morphs by determining their maintenance metabolic rates (MMR) across a range of typical field temperatures (MMR profile) in two different populations. It further tested for differences between the two colour morphs in the temperatures of the microhabitats they inhabit. If thermal niche specialization maintains the polymorphism, the morphs should display distinct thermal optima. Furthermore, in the field, the morphs should segregate into thermal niches corresponding to their particular thermal optima.

## Results

### Maintenance Metabolic Rate

Metabolic rate data, log (ln) transformed to linearize the relationship between oxygen consumption and mass [28,29], were examined using repeated-measures MANCOVA. This allowed us to test for significant influence of location, season, and phenotype on MMR and on the response profile of MMR across a thermal range. Log transformed mass was included as a covariate to appropriately adjust for variation in the size of animals [30].

**Figure 1:**
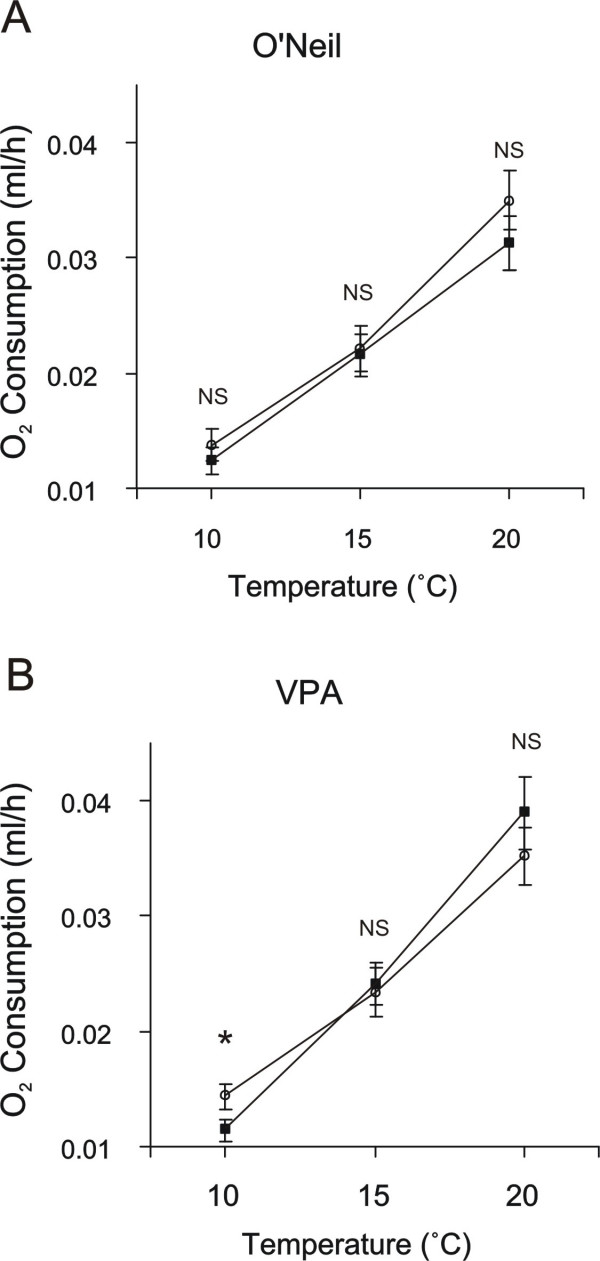
**MMR Profiles by Location and Phenotype**. MMR profiles depicting O_2 _consumption rates across temperatures for striped and black morphs for (a) O'Neil and (b) VPA. Least-Square Means (adjusted for body mass variation between groups) for whole body O_2 _consumption rate. Closed squares and open circles are black morphs and striped morphs respectively. Error bars represent ± 1 SE. An asterisk indicates a significant (p < 0.05) t-test, and NS indicates no significant difference between morphs

In the repeated measures analysis 'within individual' variation in MMR indicates variation in maintenance costs across temperatures (i.e. variation in MMR profile). Phenotype was a significant source of variation in MMR profile (temperature X phenotype effect in Table 1) indicting that phenotypes vary in their response to temperature (see Figure 1). Location was also a significant source of variation in the MMR profile primarily through its significant interaction with phenotype (temperature X location X phenotype effect in Table 1).

**Table 1:** Within Subjects Effects of Repeated Measures MANCOVA for MMR. Analysis of temperature-specific MMR profile of striped and black morphs. Analysis was performed on the natural log of O_2 _consumption rates with mass as a covariate

Source	df Num	df Den	F	*p*
temperature	2	94	176.65	<0.0001
temperature X ln mass	2	94	0.13	0.8787
temperature X phenotype	2	94	3.21	0.0448
temperature X location	2	94	2.66	0.0754
temperature X season	2	94	0.83	0.4375
temperature X phenotype X location	2	94	3.45	0.0359

**Figure 2:**
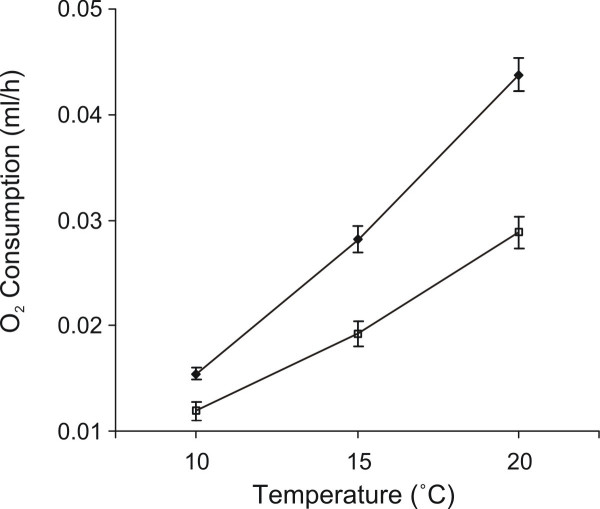
**MMR Profiles by Season**. MMR profiles depicting O_2_ consumption rates across temperatures for salamanders in the autumn and summer. Least-Square Means (adjusted for body mass variation between groups) for whole body O_2 _ consumption rates. Closed diamonds and open squares represent autumn and summer values respectively. Error bars represent ± 1 SE.

Between individuals comparisons in the repeated measures analysis tested for differences in mean MMR across all temperatures. Season was the only significant source of variation in MMR between individuals (Table 2). This indicates that the MMR profile in the autumn (Figure 2) was shifted up relative to the summer. Differences between phenotypes or locations were not significant (Table 2).

**Table 2:** Between Subjects Effects from Repeated Measures MANCOVA For MMR. Analysis of mean MMR of striped and black morphs. Analysis was performed on the natural log of O_2 _consumption rates with mass as a covariate

Source	df	Type III SS	MS	F	*p*
ln mass	1	0.5883	0.5883	1.96	0.1649
phenotype	1	0.3119	0.3119	1.04	0.3108
location	1	0.0328	0.0328	0.11	0.7416
season	1	12.4545	12.4545	41.47	<0.0001
phenotype X location	1	0.0092	0.0092	0.03	0.8611
error	95	28.5291	0.3003		

In independent contrasts for each population at each temperature, phenotype was a significant source of variation only in salamanders from VPA at 10°C (p < 0.01). The significant phenotypic influence on MMR profile (Table 1) is attributable in part to this effect (Figure 1). The mean whole body MMR (O_2 _consumption ml/h) for each morph within each location and season is listed in Table 3. Estimates of MMR (Table 3) are similar to reported estimates that have been standardized to ml/h O_2 _consumption [31] for temperatures in the 10°C, 15°C and 20°C ranges [32].

**Table 3:** MMR Means by Season, Morph and Location. Mean whole body MMR (ml/h O_2 _consumed) and Standard Errors for each morph within each season and location.

Season	Temperature (°C)	Phenotype	O'Neil	SE	VPA	SE
autumn	10	black	0.0151	0.0012	0.0143	0.0012
		striped	0.0161	0.0016	0.0165	0.0008
	15	black	0.0248	0.0022	0.0293	0.0021
		striped	0.0280	0.0029	0.0307	0.0028
	20	black	0.0386	0.0029	0.0476	0.0027
		striped	0.0426	0.0033	0.0460	0.0035
summer	10	black	0.0119	0.0017	0.0083	0.0009
		striped	0.0135	0.0023	0.0129	0.0017
	15	black	0.0207	0.0028	0.0184	0.0018
		striped	0.0190	0.0023	0.0184	0.0023
	20	black	0.0276	0.0031	0.0295	0.0044
		striped	0.0307	0.0033	0.0277	0.0018

## Field distribution across thermal environments

**Figure 3:**
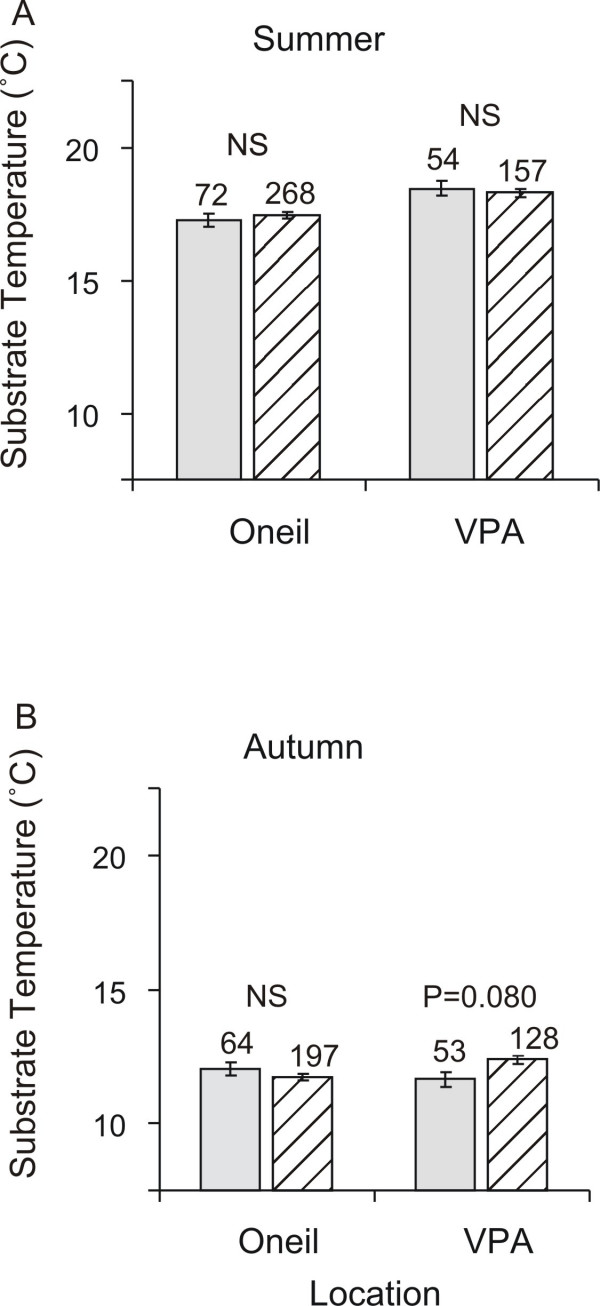
**Substrate Temperature by Location and Season**. Least-Square Means for Substrate Temperature (°C) where colour morphs were found by location in the (a) autumn and (b) summer. Solid bars are black morphs, and striped bars are striped morphs. Error bars represent ± 1 SE. Numbers above each bar indicate sample size. NS indicates no significant difference between morphs (*p *> 0.1).

Seasonal change was operationally defined by the point of temperature stabilization after a rapid and consistent decline in surface and substrate temperature across both sites for several weeks beginning 5 September and ending 19 September. Location, season and the interaction between location and season were significant sources of variation (3 way ANOVA) in the substrate temperature where the salamanders were found (Table 4). Phenotype was a significant source of variation in substrate temperature through its three-way interaction with season and location (Table 4). This significant interaction appears to be attributable in part to the marginally significant difference between the morphs from VPA in the autumn (*p *= 0.079; t-test). No differences between the morphs were detected in the other three comparisons (*p *> 0.35 in all cases; t-test). Figure 3 summarises the mean substrate temperature at which each morph within each location and season was found.

**Table 4:** ANOVA on Substrate Temperatures. ANOVA of substrate temperatures where individuals were located. Geographic location, season, morph phenotype and the interaction between those predictors were included as possible sources of variation in substrate temperature.

Source	df	Type III SS	Mean Square	F Value	*p*
location	1	59.326	59.326	14.78	0.0001
season	1	6231.869	6231.869	1553.07	<.0001
phenotype	1	2.448	2.448	0.61	0.4349
location X season	1	34.119	34.119	8.50	0.0036
location X phenotype	1	4.619	4.619	1.15	0.2836
season X phenotype	1	1.845	1.845	0.46	0.4979
location X season X phenotype	1	19.301	19.301	4.81	0.0285

## Discussion

If morphs differ in their MMR profiles, they may also differ in their net allocatable energy available for growth and reproduction, and hence in their fitness[21,25,31]. This in turn could constitute a mechanism for the maintenance of a colour polymorphism. We found that colour phenotype significantly contributes to variation in MMR profile (Table 1). This pattern of colour contributing to variation in metabolic rate and, in particular, the pattern of the black morph from VPA having a significantly lower metabolic rate than the striped morph at 10°C (Figure 1b) agrees with a study by Moreno [11], who found that the black morph had a lower SMR than the striped morph at 15°C. For thermal niche specialization to directly maintain the polymorphism however, both morphs must have a niche where they are favoured, and our study did not find evidence of this. Within the range of temperatures that we studied we did not find a consistent pattern across populations in either metabolic response or in thermal niche choice.

The data collected in this study provided the ability to differentiate morphs with even subtle differences in metabolic rate and thermal profile. While differences between morph could be detected we did not find evidence that these differences support a hypothesis that morphs are adapted for different thermal optima. We only found significant differences in MMR between the morphs at 10°C for animals from VPA and in that population there was no evidence of separate thermal niches. For separate niches to exist each morph would have to have a thermal niche in which it is favoured. This pattern of response was evident in neither the O'Neil nor VPA population. Furthermore, given that the influence of phenotype on MMR profile varied significantly between locations, thermal niche specialization is unlikely to be a uniform force directly maintaining the polymorphism in these populations. The existence of significant variation between morphs in MMR profile across temperature (Table 1), does however indicate a linkage of some sort between morph type and thermal optima. The existence of significant interactions between morph phenotype and location in the determination of thermal profile also indicated that while potentially important, any relationship between phenotype and thermal specialization is not consistent across populations.

This study demonstrates that black and striped morphs of *P. cinereus *are sometimes found in different thermal microhabitats. The two morphs seem to segregate into different thermal habitat in some locations (marginally significant for VPA in Figure 3). However, the difference is once again dependent upon the census location and season (Table 4). Differences between morphs in their thermal microhabitat distribution are consistent with data suggesting that morph frequencies can vary with air temperature [11] and with temperature proxies such as climate [7] and season [10]. Furthermore, since we find that patterns of thermal segregation are not consistent, we confirm the contradictory patterns of niche preference in these previous studies. Lotter and Scott [7] found striped morphs in lower frequencies in regions defined as "warm", and Moreno [11] found that striped morph frequency decreased with increasing temperatures. In contrast, Test [10] found that striped morph frequency increased with increasing temperatures through the summer. We conclude from these previous studies that our results accurately reflect a relationship between thermal habitat segregation and colour morph that is inconsistent from population to population.

This study, as well as Moreno's study [11] found the black morph to have a lower metabolic rate under certain conditions. Either chromosomal or pleiotropic linkage between colour phenotype and metabolism could be responsible for this relationship. If pleiotropy is responsible for the relationship between these traits then there should be a consistent relationship across all populations. However, if chromosomal linkage is responsible, linkage could be inconsistent from population to population since linkage pairing could vary from population to population. Because we find differences in the relationship between MMR and morph type between locations, it appears that chromosomal linkage is more likely to be responsible for the association of colour and metabolism than pleiotropy. Correlated response to selection on colour morphs should, in this case, vary from location to location depending on the particular linkage within a given population or region.

Alternative explanations for the maintenance of the polymorphism given evidence (here and [11]) that the black morph has at times a lower maintenance expenditure remain unexplored. Possible explanations for maintenance of such colour polymorphisms are extensive and have been thoroughly reviewed by Roulin [3] and Hoffman and Blouin[12]. Roulin has pointed out that proximate adaptive explanations for the existence of a colour polymorphism may fall into three classes: historical, direct, or indirect effects [3]. A historical association between colour and other fitness related traits, due to divergent selection during allopatry, might be carried into the present. The apparent mosaic of various associations between colour morph and metabolic rate indicate that no single historical event is responsible for the polymorphism in *P. cinereus*.

There may be some direct selection on the colour polymorphism in *P. cinereus*. Differences in solar heating are unlikely to be of any importance in this species as is common in many species [3] since the species is not active during daylight. It is possible however that colour is important in defense or mating. Such direct selection on colour may support the polymorphism or it may balance indirect selective effects due to correlated response to selection on thermal niche. The potential for selection on the all red 'eurythristic' morph as a Batesian mimic of red spotted newts has been demonstrated [33,34]. However the eurythristic form is too geographically restricted to account for the more common striped vs. black polymorphism. Striped morphs could be more cryptically coloured than the black morphs or, since predators often prey on the most common prey form, frequency-dependent selection can be maintaining the polymorphism even without mimicry [19]. No studies, however, have yet documented direct selection supporting the common striped/black polymorphism.

This and previous studies [7,10,11] have found evidence for thermal niche differentiation of the two different colour morphs. This provides the opportunity for a correlated response of to selection on temperature specific metabolic rate. While this study fails to find evidence for unique thermal optima for the two morphs over typical summer field temperatures, seasonal dynamics may provide a more complex selective regime than examined here. The differences between populations in the way in which the colour morphs respond to temperature indicate that while correlated response to selection may be an important part of the dynamics of the polymorphism it is not a consistent one. We cannot eliminate the possiblity of plastic response of metabolic rate to environmental factors upon which morphs might be segregating. This type of influence has been documented in Tawny Owls [35] although in this case we have no evidence supporting such a plastic response. We speculate insead that some sort of indirect response to selection on thermal niche is probably important in maintenance of the polymorphism, but that historical linkage associations between the morphs and the genes under selection may be locally variable.

## Conclusion

The two colour morphs of *P. cinereus *sometimes differ in their MMR profile and in the temperature microhabitat in which they are found. These differences are dependent on location and season. However these differences in MMR profile suggest that only one morph could enjoy a fitness advantage via this mechanism in these populations under the conditions studied. This is not consistent with the hypothesis that a simple correlated response to selection across multiple niches maintains the polymorphism. Instead, a correlated response to selection in conjunction with another selective regime(s) may maintain the polymorphism. Alternatively incomplete linkage between colour morph and the genes under selection may create a geographic mosaic of different correlated selective responses.

## Methods

### Site descriptions

Two study sites, 6.7 kilometres apart were used. One site is located in O'Neil Woods Metro Park (O'Neil), Bath, Ohio (N41° 10.1120, W081° 35.448) and is 1.9 km^2^. The second site, the Valley Picnic Area on Riverview Road (VPA), is located in the Cuyahoga Valley National Park, Peninsula, Ohio (N41° 13.4410, W081° 33.7020) and is 6.3 km^2^. Both sites are hilly beech-maple forests, with well-drained soil, limited underbrush, and many cover objects.

### Collection and maintenance of organisms

Animals (n = 101) from the two sites were collected for metabolic rate measurements. Salamanders were held for 5–6 days and housed individually in plastic containers with moistened paper towels. Salamanders were held at 10°C for the first 24 hours of captivity and then at the next day's test temperature for each subsequent night. Salamanders were kept on a light:dark cycle of 14:10 h for the summer trials and 12:12 h for the autumn trials. Each was fed six fruit flies (*Drosophila melanogaster*) every evening beginning two days before testing so that all animals were in a digestive state similar to free-ranging salamanders.

### Metabolic rate measures

Temperature-dependent metabolic rate was measured at 10.0°C, 15.0°C, and 20.0°C. Metabolic measurements were conducted while salamanders were resting inside respirometry chambers under full-spectrum lighting, all housed within a digitally controlled incubator. Salamanders did not have access to radiant or other external sources of heat, insuring that their body temperatures were identical to the controlled temperature of the cabinet during all trials. The order of testing for the three temperatures was randomized for each individual. Measurement of MMR, the O_2 _consumption during the scotophase (07:00 to 20:00 hours local time) of fed animals [36], allowed inclusion of relevant sources of variation such as nutritional status and variation in metabolic rate not included in SMR [37,38].

Automated closed system respirometry (Sable TR-3; Sable Systems International, Henderson, NV) was used to simultaneously record oxygen (Ametek S-3A, Pittsburgh, PA) and carbon dioxide (LiCor 6251, Lincoln, NE) levels. Five animals measured at each temperature over five evenly spaced automated cycles of 6 minutes each during any given day. The average O_2 _consumption rate (ml/h) of each animal was calculated using equations derived from Withers [39]. During the trials, the animals sat on moistened sponges to prevent dehydration in 5 cc cylindrical chambers.

### Field distributions across thermal environments

To determine correlations between temperatures and colour morph location, censuses were taken within each site. Sites were searched between June 12 and October 23, 2004. Five-hour censuses were conducted biweekly at each site by turning over potential cover objects. Data collected during each census included date, location, time, substrate temperature, temperature 5 cm above the substrate, colour phenotype, snout-vent length, and recapture status. Both substrate and air temperatures were measured simultaneously using type K thermocouples (Fluke 54 II Thermometer; 1999 Fluke Corporation, Everett, WA). Individuals (n = 993) were marked via toe clipping to prevent resampling of individuals.

### Statistical analysis

Metabolic rate data were natural log (ln) transformed to linearize the relationship between oxygen consumption and mass [28,29]. Repeated-measures MANCOVA was used to test for significant influence of location, season, and phenotype on MMR. Log transformed mass was included as a covariate to appropriately adjust for variation in the size of animals [30]. Sequential model reduction resulted in a final model that included ln mass, location, season, phenotype, and phenotype X location as sources of variation in the MMR. Inspection of model residuals gave no indication of violation of MANCOVA assumptions.

## Authors' contributions

EEP designed the study, executed the experiments, collaborated on analysis of the data and produced the first draft of the manuscript. FBGM gave advice in the design of the study, took a large role in the statistical analyses and took primary responsibility for the final manuscript. PHN provided the expertise and equipment necessary for metabolic rate measures and assisted in drafting the manuscript.
